# Content of micronutrients, mineral and trace elements in some Mediterranean spontaneous edible herbs

**DOI:** 10.1186/s13065-015-0137-9

**Published:** 2015-10-14

**Authors:** Maria Grazia Volpe, Melissa Nazzaro, Michele Di Stasio, Francesco Siano, Raffaele Coppola, Anna De Marco

**Affiliations:** Istituto di Scienze dell’Alimentazione, CNR, Via Roma 64, 83100 Avellino, Italy; Dipartimento di Agricoltura, Ambiente e Alimenti, Università degli Studi del Molise, Via Francesco de Sanctis, 86100 Campobasso, Italy; Dipartimento di Biologia, Sezione di Biologia Evolutiva e Comparata, Università degli Studi di Napoli “Federico II”, Via Mezzocannone 8, 80134 Naples, Italy

**Keywords:** Edible, Wild herbs, Mineral elements, Micronutrients, Pollution, Heavy metals, Food analysis

## Abstract

**Background:**

The analysis of mineral elements composition was determined in three wild edible herbs (*Cichorium intybus* L., *Sonchus asper* L. and *Borago officinalis*) collected in seven different sampling sites which were characterized by different pollution grade. The detection of mineral elements (Ca, K, Mg and Na), micronutrients (Cu, Fe, Li, Mn and Zn) and heavy metals (As, Cd, Hg, Ni and Pb) was performed.

**Results:**

The results obtained show that in most cases a direct relationship appeared between the amount of elements and the sampling sites. The highest concentrations of heavy metals were found in samples grown in polluted soils. These evaluations showed that contaminants in plants may reflect the environmental state in which they develop.

**Conclusion:**

The examined species are a good source of mineral elements and micronutrients, making them particularly adapt to integrate a well-balanced diet. The accumulation of heavy metals showed that contaminants in plants may reflect the environmental state in which they develop. Results showed high concentrations of heavy metals in samples taken in locations characterized by high human activity and in some samples from the local market, of which no one knows the collection area.

## Background

Wild herbs were important foods in the traditional diet of the first European farmers. Modern Mediterranean cultures still consider wild plants for nutrition, using them both raw and cooked or to prepare several traditional dishes [1–6]. The consumption of wild herbs integrates a well-balanced diet enriched with leafy green vegetables. Several wild and aromatic herbs are also used for medicinal and traditional phytotherapic purposes [7, 8], since they are considered a good source of essential minerals [4, 9–12]. Mineral elements are usually found in vegetables as constituents of bioactive molecules, and carry out important functions in the human body, as components of structural proteins, cofactors and activators of enzymes, regulators of nerve transmission, muscle contraction, osmotic pressure and salt-water balance [4].

Spontaneous herbs are also a potential link to transfer contaminants and heavy metals from environment to human through the food chain. Heavy metals such as cadmium, lead and mercury are often polluting substances present in the air as a result of different types of industrial activity. Even when their concentration in the atmosphere is low, they can accumulate in the soil entering the food chain (both by land and by water) [13–16]. Exposure to heavy metals are associated with multiple health effects, with varying degrees of severity and conditions: kidney problems and bone, neurobehavioral and developmental disorders, high blood pressure and potentially even lung cancer [13, 17–19]. Among heavy metals, As, Cd, Hg, Pb and Ni are the most important to consider in terms of food contamination [16, 20, 21], which depend on many complex factors like level and duration of contaminant exposure, agronomic management, plant genotype, stage of plant development at harvest time [16]. The major pathway of human exposure is food consumption, respect to other ways of exposure [22]. Herbal foods are natural and therefore the widespread public opinion is that they are harmless and free from adverse effects. Nevertheless, a good quality control for herbal food is important in order to protect consumers from contamination. The present work aimed at evaluating the accumulation of some mineral elements (Ca, K, Mg and Na), trace elements (Li), micronutrients (Cu, Fe, Mn and Zn) and heavy metals (As, Cd, Hg, Ni and Pb) in *Cichorium intybus* L., *Sonchus asper* L. and *Borago officinalis*, collected in selected sampling sites of the Irpinian territory (Avellino, Campania, Italy), characterized by different anthropic activities. The selected edible species were chosen because they are widely consumed in traditional meals such as i salads, soups, mixed dishes and pies. The part of the plant that is preferentially eaten is the basal leaf petioles before the plant has begun flowering or fully developed.

## Results and discussion

Table 1 shows the concentration of mineral elements in herbs for the various locations. The ranges of mineral elements concentrations were between 3417 (*C. intybus* at S-1) and 8589 mg kg^−1^ DW (*B*. *officinalis* at S-4) Ca, 26350 (*S*. *asper* at S-1) and 60,235 mg kg^−1^ DW (*S. asper* at S-5) K, 1505 (*B. officinalis* at S-7) and 5396 mg kg^−1^ DW (*B*. *officinalis* at S-3) Mg, 1242 (*S*. *asper* at S-2) and 7701 mg kg^−1^ DW (*B. officinalis* at S-3) Na.

**Table 1:** Concentration of minerals in herbs (g kg^−1^ dry weight) at various sampling sites

Sampling site	Ca (mean ± SD)			K (mean ± SD)			Mg (mean ± SD)			Na (mean ± SD)		
	*C. intybus*	*S. asper*	*B. officinalis*	*C. intybus*	*S. asper*	*B. officinalis*	*C. intybus*	*S. asper*	*B. officinalis*	*C. intybus*	*S. asper*	*B. officinalis*
S-1	3.42 ± 0.08^A,a^	3.75 ± 0.07^a^	4.14 ± 0.03^a^	42.44 ± 2.39^b^	26.35 ± 1.15^a^	37.64 ± 1.15^ab^	4.52 ± 0.01^b^	3.89 ± 0.02^ab^	3.60 ± 0.00^bc^	1.28 ± 0.24^a^	2.77 ± 0.27^a^	1.75 ± 0.20^a^
S-2	4.67 ± 0.30^a^	3.45 ± 0.65^a^	7.57 ± 0.65^b^	30.46 ± 0.49^a^	30.61 ± 0.29^a^	36.90 ± 0.29^ab^	4.53 ± 0.01^b^	3.68 ± 0.01^a^	4.91 ± 0.00^c^	1.37 ± 0.06^a^	1.24 ± 0.01^a^	3.73 ± 0.04^b^
S-3	4.78 ± 0.74^a^	5.35 ± 0.80^ab^	6.85 ± 0.80^ab^	47.45 ± 2.36^b^	39.50 ± 1.97^ab^	33.33 ± 1.97^a^	3.65 ± 0.01^b^	4.92 ± 0.02^b^	5.40 ± 0.01^c^	4.21 ± 0.19^b^	4.10 ± 0.60^b^	7.70 ± 0.15^c^
S-4	5.66 ± 0.10^ab^	4.44 ± 0.03^a^	8.59 ± 0.03^b^	37.08 ± 0.90^a^	34.49 ± 1.56^a^	40.22 ± 1.56^b^	3.48 ± 0.01^b^	3.66 ± 0.01^a^	4.94 ± 0.01^c^	2.38 ± 0.08^a^	2.29 ± 0.05^a^	2.33 ± 0.13^a^
S-5	7.25 ± 0.06^b^	6.60 ± 0.02^b^	7.07 ± 0.02^ab^	29.68 ± 0.80^a^	60.24 ± 0.88^c^	26.43 ± 0.88^a^	4.23 ± 0.01^b^	4.51 ± 0.01^b^	4.34 ± 0.01^bc^	7.21 ± 0.13^c^	3.13 ± 0.01^b^	3.78 ± 0.06^b^
S-6	6.95 ± 0.80^b^	6.70 ± 0.76^b^	7.66 ± 0.76^b^	33.57 ± 1.77^a^	42.54 ± 2.09^b^	42.02 ± 2.09^b^	2.84 ± 0.00^a^	3.16 ± 0.02^a^	1.91 ± 0.01^a^	2.00 ± 0.02^a^	2.03 ± 0.03^a^	2.21 ± 0.02^a^
S-7	7.24 ± 0.35^b^	7.58 ± 0.53^b^	7.25 ± 0.53^b^	32.45 ± 0.71^a^	56.01 ± 1.86^c^	55.54 ± 1.86^c^	2.55 ± 0.01^a^	3.31 ± 0.01^a^	1.50 ± 0.01^a^	2.56 ± 0.03^a^	3.13 ± 0.30^b^	2.91 ± 0.13^a^

^A^In each column, different letters indicate significant differences (p < 0.05) for each category

The trace elements levels (Table 2) were established between 4.10 (*S. asper* at S-7) and 19.85 mg kg^−1^ DW (*B. officinalis* at S-4) Cu, 11.55 (*B. officinalis* at S-6) and 120.40 mg kg^−1^ DW (*C. intybus* at S-1) Fe, 5.48 (*C. intybus* at S-1) and 68.76 mg kg^−1^ DW (*B. officinalis* at S-4) Li, 7.98 (*S. asper* at S-7) and 47.06 mg kg^−1^ DW *(B*. *officinalis* at S-4) Mn, 27.30 (*C. intybus* at S-3) and 84.54 mg kg^−1^ DW (*B. officinalis* at S-6) Zn.

**Table 2:** Concentration of trace elements in herbs (mg kg^−1^ dry weight) at various sampling sites

Sampling site	Cu (mean ± SD)			Fe (mean ± SD)			Li (mean ± SD)			Mn (mean ± SD)			Zn (mean ± SD)		
	*C. intybus*	*S. asper*	*B. officinalis*	*C. intybus*	*S. asper*	*B. officinalis*	*C. intybus*	*S. asper*	*B. officinalis*	*C. intybus*	*S. asper*	*B. officinalis*	*C. intybus*	*S. asper*	*B. officinalis*
S-1	14.53 ± 0.32^A,b^	6.08 ± 0.08^a^	8.47 ± 0.32^ab^	120.40 ± 2.67^c^	43.35 ± 0.32^b^	91.84 ± 7.83^c^	5.48 ± 0.33^a^	19.07 ± 0.45^a^	18.79 ± 0.20^a^	19.95 ± 0.16^a^	27.53 ± 0.40^ab^	25.34 ± 0.40^a^	65.28 ± 1.78^b^	54.38 ± 0.73^ab^	50.82 ± 1.70^ab^
S-2	6.21 ± 0.24^a^	5.39 ± 0.08^a^	7.64 ± 0.24^a^	49.70 ± 2.75^b^	28.63 ± 0.89^a^	77.99 ± 2.26^c^	39.09 ± 0.79^ab^	24.77 ± 0.63^ab^	52.12 ± 0.82^bc^	19.60 ± 0.89^a^	19.11 ± 0.24^a^	40.00 ± 1.13^b^	73.15 ± 1.29^b^	31.22 ± 0.40 ^a^	59.18 ± 1.86^b^
S-3	5.67 ± 0.08^a^	8.40 ± 0.02^ab^	8.40 ± 0.16^ab^	43.05 ± 0.81^b^	42.19 ± 0.81^b^	66.36 ± 1.53^b^	47.11 ± 0.35^b^	38.11 ± 0.57^b^	57.25 ± 0.50^c^	26.11 ± 0.24^ab^	35.13 ± 0.24^b^	29.93 ± 0.08^ab^	27.30 ± 0.57^a^	78.85 ± 1.21^b^	48.30 ± 0.24^a^
S-4	8.73 ± 0.65^a^	10.43 ± 0.08^ab^	19.85 ± 0.48^b^	58.14 ± 2.58^b^	51.80 ± 2.18^b^	75.60 ± 2.67^c^	35.28 ± 0.68^ab^	29.70 ± 0.61^b^	68.76 ± 0.86^c^	22.44 ± 0.97^a^	16.59 ± 0.40^a^	47.06 ± 0.32^b^	45.68 ± 1.05^ab^	57.89 ± 1.05^ab^	53.74 ± 0.08
S-5	5.57 ± 0.16^a^	6.30 ± 0.40^a^	6.48 ± 0.16^a^	46.12 ± 1.05^b^	40.78 ± 0.73^b^	69.13 ± 0.16^b^	56.60 ± 2.44^b^	35.56 ± 0.79^b^	48.82 ± 0.43^b^	35.54 ± 0.08^b^	24.85 ± 0.24^ab^	33.08 ± 0.08^ab^	52.26 ± 0.08^ab^	55.91 ± 0.24^ab^	41.30 ± 0.81^a^
S-6	11.72 ± 0.73^b^	16.84 ± 0.81^b^	9.87 ± 0.40^ab^	18.84 ± 2.75^a^	32.23 ± 0.16^a^	11.55 ± 2.34^a^	9.38 ± 0.22^a^	12.71 ± 0.14^a^	22.84 ± 0.43^a^	23.81 ± 1.62^a^	29.94 ± 1.05^b^	33.85 ± 1.70^ab^	74.16 ± 4.85^b^	66.95 ± 2.50^b^	84.54 ± 3.47^c^
S-7	6.54 ± 0.06^a^	4.10 ± 0.08^a^	5.57 ± 0.08^a^	52.78 ± 1.94^b^	34.97 ± 0.73^a^	47.17 ± 5.09^b^	14.33 ± 0.25^a^	9.42 ± 0.14^a^	25.90 ± 0.29^a^	20.63 ± 0.16^a^	7.98 ± 0.08^a^	21.48 ± 0.24^a^	65.95 ± 0.73^b^	47.57 ± 0.40 ^a^	41.27 ± 0.16^a^

^A^In each column, different letters indicate significant differences (p < 0.05) for each category

Data showed that K was the most abundant mineral element in the evaluated herbal species.

The highest content of K was measured in *S. asper* at S-5, and in *S. asper* and *B. officinalis*, both from S-7. However, every single species showed variable K among the various sampling sites. The levels of Na were higher at S-3 and at S-5, in comparison with the herbs collected in the remaining sites. Furthermore, the values were similar in samples within the same area, in spite of some higher values (*B. officinalis* at S-3 and *C. intybus* at S-5) that were measured. The lowest levels of Ca were found in the herbs purchased at S-1, as well as in *S. asper* collected at S-2. With the exception of a few peaks measured (*B. officinalis* at S-2, S-3, and S-4), the content of Ca was similar among the three species, in the same sampling area. Samples collected at S-6 and S-7 were similar in Mg values, and both were lower than those measured in the other sites. The levels of Cu were similar among the herbs collected in the same area, and only a few peak values were observed (*C. intybus* at S-1, *B. officinalis* at S-4, and *S. asper* at S-6). The herbs from S-4 and S-6 showed higher Cu content than the other samples. The lowest concentrations of Fe were measured in *C. intybus* and *B. officinalis*, both from S-6. In comparison to these latter species, *S. asper* had a smaller range of Fe content. Mn and Zn levels had variable values in the different sites, for each monitored species. The lowest concentrations of Li were measured at S-1, S-6 and S-7. In 5 of 7 sampling sites, *S. asper* contained less Fe than *C. intybus* and *B. officinalis*.

There is scant information on the composition of the monitored selected herbs. Medrano et al. [3] referred the following values of mineral elements constituents in *B*. *officinalis*: 68,000 mg kg^−1^ DW K, 12,000 mg kg^−1^ DW Na, 11,000 mg kg^−1^ DW Ca, 2100 mg kg^−1^ DW Mg, 200 mg kg^−1^ DW Fe, 36 mg kg^−1^ DW Mn, 23 mg kg^−1^ DW Zn, and 15 mg kg^−1^ DW Cu. Nevertheless, our results are in accordance or slightly lower than the mean values of mineral elements and trace elements reported in literature in edible herbs used as spices and condiments [4, 9, 10].

Overall, a relationship appeared between the concentration of mineral elements and the sampling locations, since mineral elements levels were often similar among the species collected in the same site. If this relation was not evident, we supposed that the uptake of mineral elements was influenced by the plant genotype or by the stage of development, which are factors that can affect the characteristics of plants, since we must consider that samples were randomly collected.

The level of micronutrients, mineral and trace elements in plants is conditional, the content being also affected by chemical and physical properties of soil, such as pH and presence of organic matter, and by the ability of plants to selectively accumulate some of these elements. Further possible causes of variation in mineral elements content would include agricultural practices, rainfall and temperature. Previous studies report different content of micronutrients, mineral and trace elements in commercial leafy vegetables, such as *Spinaciaoleracea* [23, 24] and *Brassica oleracea* var. *acephala* [24, 25] when the same species are grown in different soils.

The level of micronutrients did not seem to be influenced by the environmental status of the sampling sites. Previous studies found higher concentration of Cu and Zn in edible vegetables grown in spiked soils [26], in contaminated sites located in urban areas [27], in industrial areas [28], or in mining areas [29], in comparison with products grown in uncontaminated sites. Many studies highlighted differences in sensitivity between different crop types, demonstrating for some of them high levels of uptake, while for other restrictive behaviour depending by several factors, including bioavailability of the mineral elements in soil, crop type and metal dislocation in the crop.

**Fig. 1:**
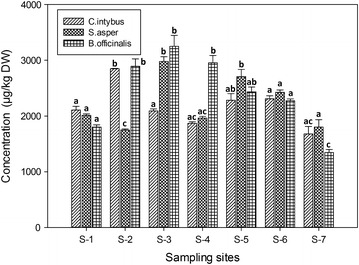
Concentration of As in three different wild herbs (mean ± SD) for each sampling site; for each wild herb *different lower case letters* indicate significant (p < 0.05)

**Fig. 2:**
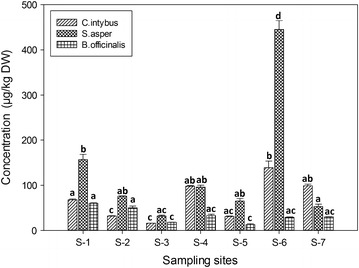
Concentration of Cd in three different wild herbs (mean ± SD) for each sampling site; for each wild herb *different lower case letters* indicate significant (p < 0.05)

**Fig. 3:**
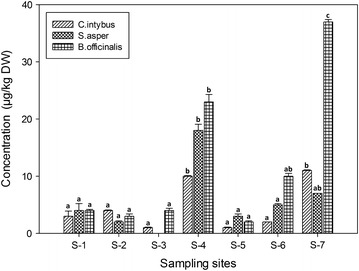
Concentration of Hg in three different wild herbs (mean ± SD) for each sampling site; for each wild herb *different lower case letters* indicate significant (p < 0.05)

**Fig. 4:**
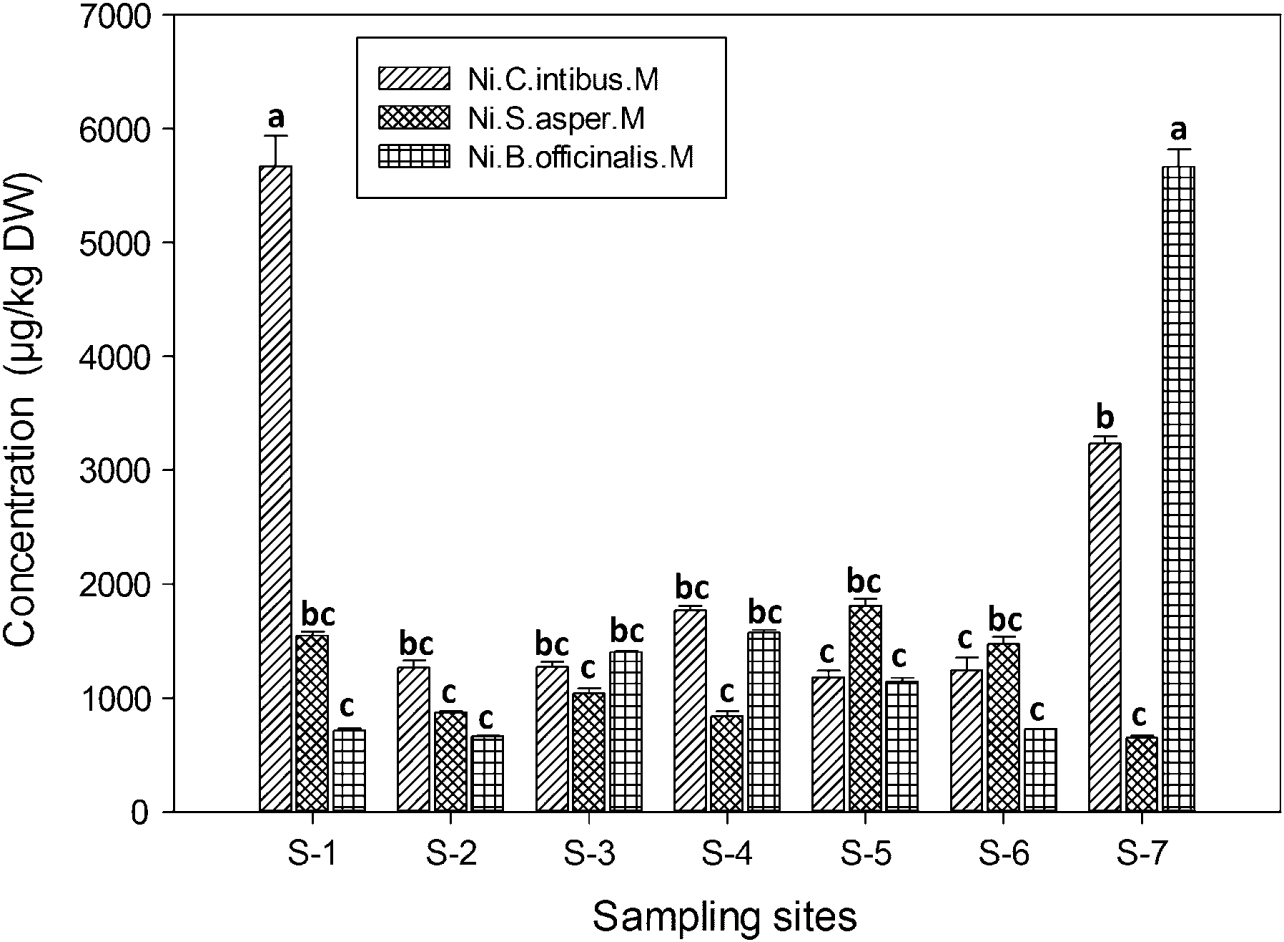
Concentration of Ni in three different wild herbs (mean ± SD) for each sampling site; for each wild herb *different lower case letters* indicate significant (p < 0.05)

**Fig. 5:**
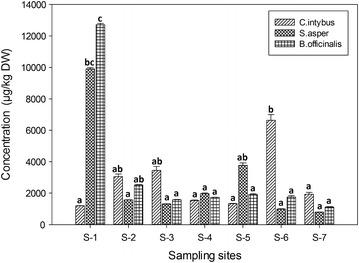
Concentration of Pb in three different wild herbs (mean ± SD) for each sampling site; for each wild herb *different lower case letters* indicate significant (p < 0.05)

Figures 1, 2, 3, 4 and 5 show the levels of heavy metals expressed as µg kg^−1^ DW. The levels of As ranged between 1346 µg kg^−1^ DW (*B. officinalis* at S-7) and 3251 µg kg^−1^ DW (*B. officinalis* at S-3).The highest concentration of Cd was 445 µg kg^−1^ DW (*S. asper* at S-6), whilst other values ranged from 13 µg kg^−1^ DW (*B. officinalis* at S-5) to 157 µg kg^−1^ DW (*S. asper* at S-1). Except for *S. asper* at S-3, all analyzed samples contained detectable Hg levels, which varied between 1.0 µg kg^−1^ DW (*C. intybus* at S-3 and S-5) and 37 µg kg^−1^ DW (*B. officinalis* at S-7).

Ni amounts ranged from 664 µg kg^−1^ DW (*B. officinalis* at S-2) to 5671 µg kg^−1^ DW (*C. intybus*at S-1). Pb content ranged from 793 µg kg^−1^ DW (*S. asper* at S-7) to 12,708 µg kg^−1^ DW (*B. officinalis* at S-1).

Metal contaminants in soils could possibly affect human health through a variety of pathways. This study also focused on the potential pathway of consumption of three wild edible herbs grown on different soils. We considered some locations as potentially polluted, in which the levels of some monitored elements resulted more elevated. In fact, the highest levels of Hg were found in the samples collected at S-4 and S-7, that corresponded to the waste collection area and to the industrial area, respectively. High amounts of Ni were also established in *C. intybus* and *B. officinalis* from S-7. Elevated levels of Cd and Pb were found at S-6, where it is reasonable to assume the presence of increased contamination of soil and atmosphere by these elements from exhaust gases of vehicular traffic. According to Nabulo et al. [30] Pb and Cd concentration in leafy vegetables decreased with increasing distance from the road edge which was characterized by a high rate of traffic. All samples in this study exhibited concentrations of Pb that are higher than Cd, and this is considered a normal situation for plants [31]. The content of As was comparable in polluted as in uncontaminated sampling sites, with the exception of the industrial zone, where the values were slightly lower. S-2 and S-3 were practically considered unpolluted areas, in which herbal samples were collected in the fields away from the town. Just as we expected, in these areas the level of toxic elements was relatively low, owing to the absence of anthropogenic activities. However, in order to explain the high levels of Cd, Pb and Ni detected in samples bought at the local market, we could hypothesize that herbs were harvested in contaminated sites, since their collection area was unknown. There are two routes where vegetation was contaminated by heavy metals, one from soil sources via root uptake [32, 33], and the other from aerial deposition onto plant leaves. Yakupoğlu et al. [34] observed that *C. intybus* collected in areas away from roads and vehicular traffic were not free of Pb, so the consumption of these plants could bring a certain amount of Pb into food chain.

In this work the levels of Cd, Pb and Ni were below the values referred by previous studies.

According to Shallari et al. [35] *C. intybus* had 1 mg/kg DW Cd, 17 mg kg^−1^ DW Ni, and 35 mg kg^−1^ DW Pb, when it grown on a soil contained high levels of metals. Previous studies on *S. asper* reported Pb contents of 2194 mg kg^−1^ DW [35] and 39 mg kg^−1^ DW [26], in samples collected in a mining area and from a site contaminated by metals. Concentration in plants from non polluted sites are indicated as 5 mg kg^−1^ DW and 1 mg kg^−1^ DW for Pb and Cd, respectively [29, 36], while the common suggested Ni concentration in vegetables varies between 0.2 and 3.7 mg kg^−1^ DW [37].

FAO/WHO [38], in terms of provisional tolerable weekly intake (PTWI) values for kg body weight, fixed the levels of safe exposure for cadmium (7 µg), lead (25 µg) and inorganic arsenic (15 µg) [39]. It is also important to determine the content of toxic element linked organically to herbal foods and presumably not assimilated via the gastro-intestinal tract.

On the other hand, the herbal species investigated in this paper aren’t eaten on a daily basis, so they should not be a major source of heavy metals.

## Experimental

### Chemicals

Methanol and nitric acid (65 %) were obtained from Sigma-Aldrich (Steinheim, Germany). Hydrogen peroxide (30 %) came from Carlo Erba Reagents (Milan, Italy). Calcium, sodium, potassium and magnesium standards were obtained from SpectroPure (Arlington, TX, USA) and lanthanum chloride was from Acros Organics (Geel, Belgium). Mercury, nickel, lead, arsenic, cadmium, copper, iron, manganese and zinc standards were purchased from Perkin Elmer (Boston, MA, USA). The water used in these experiments 18.2 MOhm cm^−1^ was purified with Milli-Q plus 185 system associated with an Elix 5 pre-system. (Millipore, Bedford, MA, USA).

### Sampling and pretreatment

**Fig. 6:**
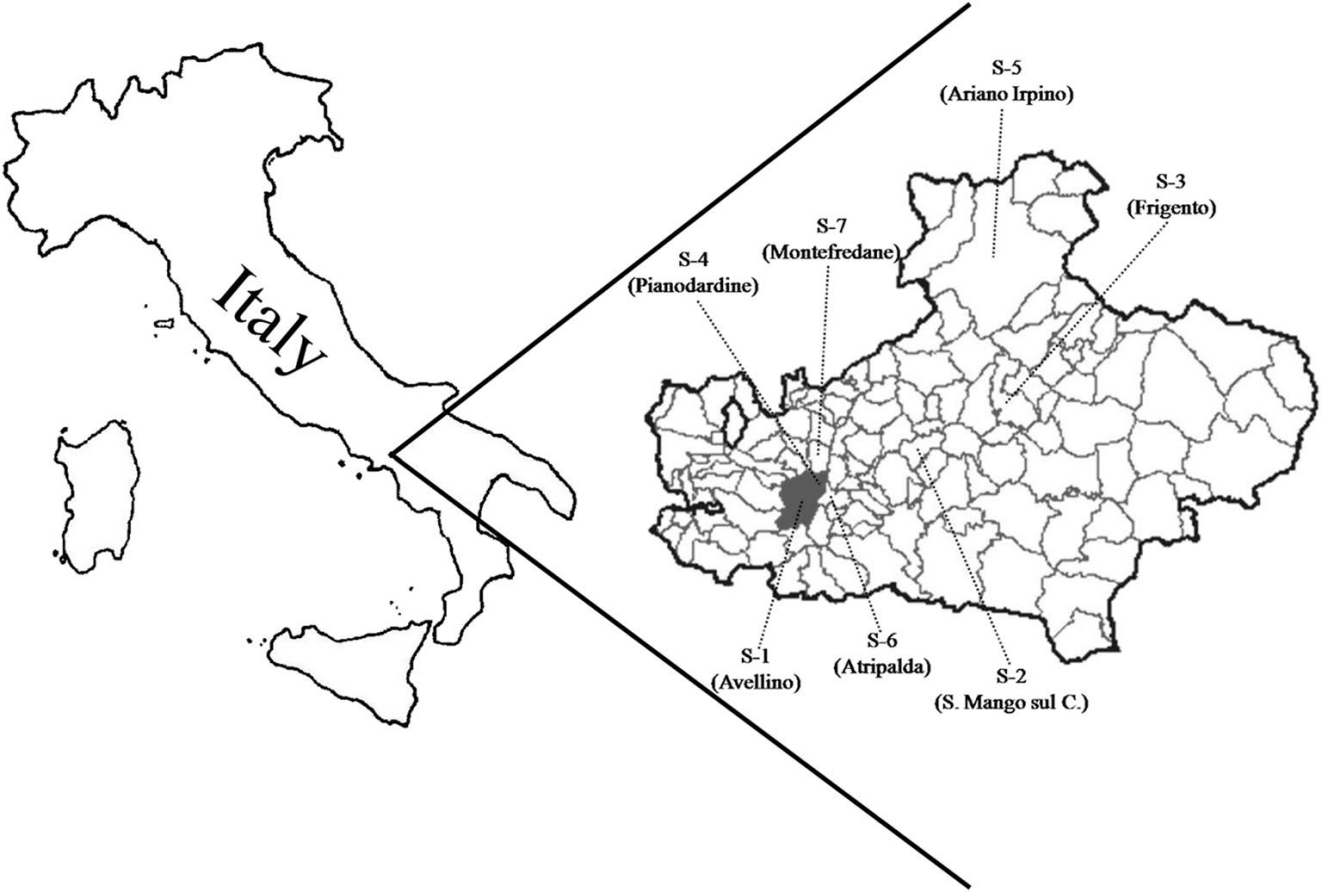
Geographical area sampling of analyzed herbs

Samples of *C*. *intybus* L. (Compositae family), *S*. *Asper* L. (Compositae family) and *B*. *officinalis* (Boraginaceae family) were collected during the spring season in seven different sites in the province of Avellino (Campania, Italy). At first, herbs were purchased in a local market of Avellino (S-1). Moreover, samples were collected in the field in six sites that were characterized by different degrees of industrial or anthropogenic impact. Specifically, two sampling sites were considered unpolluted: S. Mango sul Calore (S-2), near the town forest, and Frigento (S-3), in fallow fields away from the town. Four sampling areas were considered as a high human impact: Pianodardine (S-4), near the waste collection center of Avellino; Ariano Irpino (S-5), near the landfill; Atripalda (S-6), adjacent to the local highway; Montefredane (S-7), at the industrial area (Fig. 6). Herbs were collected in the field with a random sampling procedure. At the laboratory, the herbs were washed in fresh running water to eliminate dust, dirt, possible parasites or their eggs; after, they were again washed with deionized water. Only the edible leaf tissue was used for analyses.

### Sample digestion

The samples were weighed to determine their fresh weight and were then oven-dried at 105 °C for 72 h to determine the dry weight. The dry samples were crushed in a mortar to a fine powder. The digestion was carried out according to the method reported by Demirel et al. [40]. To one gram of dried sample, 16 mL of HNO_3_/H_2_O_2_ (6/2, v/v) of solution were added. The mixture was heated to 130 °C until the solution became transparent. After cooling, the solution was filtered and diluted to 25 mL in a volumetric flask.

### Measurement

The content of Ca, K, Li, Mg and Na was detected by Flame Atomic Absorption Spectrophotometry (F-AAS), using a Varian-Spectr AA 200 spectrophotometer (Varian, Palo Alto, CA, USA). The quantitative determinations were carried out by calibration curves using standard solutions in which the elements were in optimal concentration ranges. Lanthanum chloride (0.5%, w/v) was added to both samples and standard solutions to avoid chemical interferences, as referred by Kawashima and Valente-Soares [25]. Results were expressed as mg element/kg dry weight.

Micronutrients (Cu, Fe, Mn, and Zn) and heavy metals (As, Cd, Hg, Ni, and Pb) were detected by an inductively coupled plasma mass spectrometry, using Elan 9000 ICP-MS (Perkin Elmer, Boston, MA, USA) with Asx 520 autosampler. A calibration curve was constructed using three standard solutions for each element. Results for essential elements were expressed as mg element kg^−1^ dry weight. Results for heavy metals were expressed as μg element kg^−1^ dry weight.

Accuracy was checked by concurrent analysis of standard reference material from the Community Bureau of reference of the Commission of the European Communities (BCR no. 142R, sandy-loam soil) [40], recoveries ranged from 86 to 98 %.

### Statistical analysis

A statistical analysis was carried out using the Statistica 8.0 statistical package (Statsoftinc, Tulsa, OK, USA). The essential difference in accumulation of metals in three wild edible herbs collected in seven different sampling sites, were calculated using One-way Analyses of Variance (ANOVA), where P values are considered significant when lower than 0.05; when significant effects occurred, Duncan’s post-hoc test was performed. Prior to analysis data was tested for normality using Cochran’s test, and for homogeneity of variance using Shapiro–Wilk’s test.

## Conclusions

In conclusion, the examined species are a good source of micronutrients, mineral and trace elements, making them particularly adapt to integrate a well-balanced diet. The accumulation of heavy metals showed that contaminants in plants may reflect the environmental state in which they develop. Results showed high concentrations of heavy metals in samples taken in locations characterized by high human activity and in some samples from the local market, of which no one knows the collection area.
